# Medicine

**Published:** 1893-06

**Authors:** R. Shingleton Smith


### Progress of the Medical Sciences.

### MEDICINE.

Our views on the subject of the treatment of enteric fever appear to be undergoing a considerable change. For many years past it has been assumed that, although the pyrexia might be modified and controlled, the morbid process must run its course, and could not be either arrested or materially modified by any medicinal means. Recent discoveries on the etiology of the disease give grounds for more hopeful expectations with regard to the results of treatment. The bacillus, discovered by Koch and Eberth in 1880, whose appearance and mode of growth are now familiar to all pathologists, is generally admitted to be the actual cause of the chain of phenomena known as typhoid fever; nevertheless this bacillus is so frequently found in the soil, particularly in the autumn season, also in water, and even in the human intestines, that the bacillus itself must be harmless excepting under particular conditions. Dr. M. A. Boyd, of Dublin,[1] believes that, amongst the conditions which favour acute infection of the intestinal glands, the autumnal gastro-intestinal catarrh is perhaps the most important. Murchison believed in such a gastro-enteric catarrh as a usual precursor of enteric, and Dr. Boyd suggests that such a catarrh, in addition to being brought about by atmospheric changes or by food, may be also produced by absorption of the chemical products of the typhoid bacilli growing in the intestinal contents when present in large numbers in either food or drink. Recent bacteriological work tends to show that the entire process, so far as the typhoid bacillus is concerned, is over in fourteen days; after this period a new set of enemies arrives, and the subsequent changes which so prolong the disease are due to suppurative micrococci and their toxines. These two factors, the preliminary catarrh and the secondary phenomena of septic poisoning, lead to important practical deductions as regards treatment. They may be considered under the two headings of diet and antiseptics.

The dietetic treatment of enteric fever is chiefly of importance in connection with the etiology of the disease and relapse; a paper by Dr. Wallace Beatty[2] sums up what is known as to the connection between diet and relapse, and emphasises the necessity for a limitation in quantity of food. We are glad to find that he adopts what is commonly considered to be the best and safest food; viz., milk and milk only, diluted with a little soda water, lime water, or plain water. He considers boiling to be unnecessary, except when diarrhœa is present: we always prefer to have town milk boiled, as this is the only safeguard against organic poisons. He adds that "sometimes it is necessary to peptonise the milk": we would prefer to say that peptonising is a necessity in all severe cases where the digestive power is failing and there is a tendency to fermentative distension. He objects to farinaceous foods, on the ground that they may cause troublesome flatulence, and further that they are not needed when sufficient milk is taken. He admits beef-tea and beaten-up eggs in cases where the patient is unable to take a sufficient quantity of milk. As regards the quantity necessary, his opinion is that two to three pints of liquid nourishment in the twenty-four hours is an ample supply of food for adults, and that avoidance of excess of liquid food is the best means of counteracting diarrhœa. In the discussion following Dr. Beatty's paper, Dr. Moore stated his belief that once the specific origin of the disease was admitted, no mere error in diet could bring about a true relapse, although it might be a contributory cause of such an accident.

[1] *Trans. Roy. Acad. Med., Ireland*, vol. x., 1892. [2] *Ibid.*

Of the numerous antiseptic drugs which have been more or less successfully used for disinfection of the intestinal tract, carbolic acid appears likely to take the lead. Dr. Gramshaw[1] obtained excellent results with liquid carbolic acid and iodine; and now carbolic acid in pills is found by Dr. Charteris[2] to give results which are equally good. Each pill contains 2½ grs. of pure acid, made up with some innocuous powder and covered with keratin; the pill accordingly does not dissolve in the stomach, and will not be likely to irritate or cause sickness. On solution of the pill in the alkaline fluid of the jejunum the carbolic acid will at once begin to produce an antiseptic effect at the part where it is most likely to be needed and to be of greatest utility.

The combination of phenol with salicylic acid is also coming into favour; salol becomes decomposed in the alkaline contents of the small intestine, where the phenol may exert its usual influence.

Dr. A. M. Anderson, of Dundee, gives an account[3] of twelve cases treated with salol, and he asserts that the course of the disease is arrested on the fifth day of treatment; ten of the patients were convalescent on the fifteenth day of the disease. From the condition of the stools and the permanent suppression of the typhoid symptoms on the fifth day, Dr. Anderson infers the complete destruction of all the specific typhoid poison in the patient's body; he believes that the excreta, before being expelled, are rendered perfectly harmless and incapable of propagating the disease. These results were obtained with doses of ten grains, given every two hours.

Dr. W. C. Cahall[4] obtained excellent results from salol in doses of three grains every two hours. In sixteen cases of undoubted typhoid there was amelioration of all the symptoms; no alcohol was needed, no death occurred, and the average duration was seventeen days.

Dr. Burney Yeo,[5] whilst strongly urging the necessity for and utility of intestinal and general antiseptic treatment, prefers his own method by chlorine, and reports some excellent results therefrom.

It remains to be proved what drug will give the best results, but there is ample evidence before us that the so-called "expectant" method must no longer be tolerated. The policy of acting as if we had reached the ultimate goal of knowledge, with its lamentable results in a disease like typhoid, which as yet is ever with us in all large cities, needs still the stimulating presence of the proposed British Institute of Preventive Medicine, whose Council sends an urgent appeal for liberal support towards a great national necessity for which a large sum is required: in order that it may be carried out on an

[1] *Lancet*, June 23, 1888. [2] *Brit. Med. Journ.*, Dec. 31, 1892.
[3] See *Med. Chron.*, March, 1893.
[4] *Med. News*, Nov. 8, 1890. [5] *Treatment of Typhoid Fever.* 1892.

adequate scale, it is estimated that at least a hundred and twenty thousand pounds will be needed.

The utility of, and necessity for, antiseptic inunctions in scarlatina have been emphasised by Dr. Curgenven, whose pamphlet was reviewed in the *Journal* for December, 1892. He urges the inunction of some antiseptic volatile oil as a means of obviating the danger of infection during the desquamative stage, and asserts that this process has a specific influence on the course of the disease.

Whether one particular kind of volatile oil or antiseptic ointment is better than another remains to be proved, but it has been abundantly demonstrated that some such antiseptic application to the desquamating skin fixes or destroys the poison, and so prevents the dissemination of the disease. We need no longer expect other cases of scarlatina after the time for danger from exposure to a common cause has passed.

As regards the period of incubation and infectivity, the Clinical Society's Report [1] is a valuable contribution, which bears the stamp of considerable authority, and may be looked upon as our most recent and trustworthy guide. An abstract of this Report appears in the *British Medical Journal*, May 6, 1893, p. 958.

Everyone knows that most cases of chlorosis may be easily cured by the administration of one or other of the numerous preparations of iron. Dr. Ralph Stockman has discussed at some length the theories of its *modus operandi*.[2] Some of his conclusions are of considerable interest. He gives abundant evidence that iron given hypodermically will cure chlorosis, but he adds that no thoroughly satisfactory preparation for hypodermic use has yet been proposed, and by observations with sulphide of iron he disproves Bunge's theory that inorganic iron is useful simply by absorbing sulphuretted hydrogen in the bowel. He shows that bismuth is quite as capable as iron of doing this, but that in no case is improvement in the blood condition manifest until iron has been substituted for it. From experiments with arsenic, he considers that this drug is not in any sense a hæmatinic, and that it has no direct influence on the blood in chlorosis. He has never seen the slightest improvement from the use of manganese or of hydrochloric acid. Sir Andrew Clark's method of treatment by saline aperients gave very unsatisfactory results when the iron was omitted from the mixture, but the addition of the iron caused rapid improvement in all the cases. Dietetic treatment and rest gave results showing that, whilst

[1] *Transactions*, vol. xxv.
[2] *Brit. Med. Journ.*, April 29, May 6, 1893.

they naturally contribute greatly to bodily comfort and well-being, they cannot be regarded as a specific for the cure of anæmia, or more valuable in it than in many other morbid conditions.

As regards the best form of administration and the dose of iron, no proof has yet been given that any one preparation of iron cures chlorosis more quickly than another, and the most rational course appears to be to use those preparations which disturb digestion least. Reduced iron and freshly-prepared carbonate have been in favour of late. There appears to be little doubt that the ordinary inorganic preparations of iron cure much more quickly than organically-combined iron does. Patients fed on even a rich and varied diet, containing plenty of organic iron, do not, as a rule, recover until inorganic iron is administered. This is one of the great problems yet to be solved, why the patient with chlorosis should not be able to renew the iron of the blood from the abundant supply in the food.

This paper gives overwhelming evidence that inorganic iron is the specific for the chlorotic variety of anæmia, and that no other drug is either useful or needful; but the author's theories are not yet quite confirmed.

Further points bearing on anæmia of other kinds have been commented upon by Smart,[1] who found that arsenic, given alone, did not appear to exert any direct or appreciable effect on the renewal of the red blood corpuscles and hæmoglobin in cases of symptomatic anæmia and chlorosis, but, when combined with iron, it powerfully augmented the hæmatinic action of this drug. In pernicious anæmia full doses produced a most beneficial effect, although iron, "long and freely exhibited" had produced no appreciable result. He emphasises the fact that chlorosis may be regarded as a hæmoglobin anæmia, whilst pernicious anæmia may be looked upon as a corpuscular anæmia.

R. SHINGLETON SMITH.

